# Phototherapeutic β‑Cyclodextrin-Branched Polymer Releasing Nitric Oxide with Fluorescent Self-Reporting and Its Combination with Doxorubicin

**DOI:** 10.1021/acs.biomac.6c00462

**Published:** 2026-04-15

**Authors:** Marta Perez-Lloret, Cristina Parisi, Francesca Laneri, Gian Marco Leone, Cristina Barbagallo, Katia Mangano, Ferdinando Nicoletti, Szabolcs Béni, Milo Malanga, Salvatore Sortino

**Affiliations:** † PhotoChemLab, Department of Drug and Health Sciences, 9298 University of Catania, I-95125 Catania, Italy; ‡ Department of Biomedical and Biotechnological Sciences, University of Catania, I-95125 Catania, Italy; § Department of Analytical Chemistry, Institute of Chemistry, H-1117 Budapest, Hungary; ∥ CarboHyde Ltd., Berlini u. 47-49, H-1045 Budapest, Hungary

## Abstract

In this contribution, we report the design, synthesis, photochemical characterization, and biological validation of a novel, photoresponsive β-cyclodextrin branched polymer (**Poly-βCD1**) and its nanoassembly with doxorubicin (**DOX**). This tailored polymer covalently integrates multiple bichromophoric dyads based on nitroaniline- and coumarin-derived motifs within its macromolecular scaffold. Excitation of the **Poly-βCD1** with visible blue light results in the generation of nitric oxide (NO) from the nitroaniline moiety, and the parallel restoration of the typical emission of the coumarin fluorogenic unit, initially suppressed by Förster Resonance Energy Transfer (FRET), which acts as an optical self-reporter for the photoreleased NO. These photochemical properties are enhanced relative to those of the isolated monomer **βCD1**, synthesized as a model compound, and are well preserved after **DOX** entrapment within the polymeric network. This feature enables real-time optical monitoring of the NO photodelivery in cancer cells using fluorescence microscopy. Preliminary toxicity experiments carried out with **DOX**-sensitive and **DOX**-resistant cancer cells demonstrate that **Poly-βCD1** is well tolerated in the dark but induces cell death under light irradiation. Besides, the negligible cytotoxic action of **DOX**, used well below the therapeutic doses, alone or in combination with the polymer in the dark, is enhanced in both cell lines under light irradiation exclusively when the drug is combined with **Poly-βCD1** as a result of the combined action of NO.

## Graphical abstract

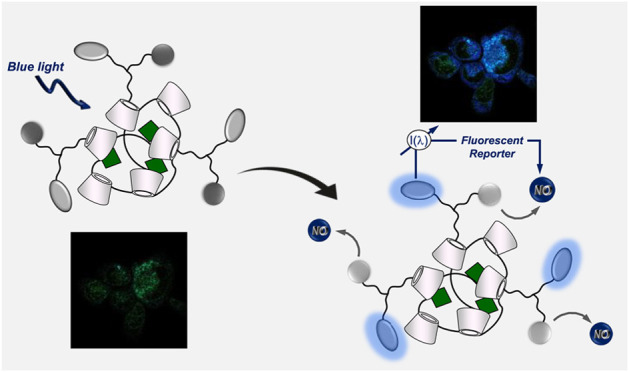

## Introduction

Polymer therapeutics represent a new class of chemical entities that have strongly contributed to the first generation of nanomedicines.^1–4^ These polymeric systems encompass drug-polymer and protein–polymer conjugates, and other nanosystems, which differ from conventional formulations designed for drug entrapment or solubilization, without resorting to chemical conjugation.^5–7^ In this context, light-activatable polymer therapeutics are particularly appealing. This is due to light’s unique features as a noninvasive tool in the emerging field of photopharmacology for controlling the release of specific therapeutic agents with very high spatiotemporal precision and, in several cases, for combining therapeutic release with fluorescence signals for cell tracking.^8–11^

Cyclodextrin (CD) branched polymers have emerged rapidly over the past two decades. CDs are cyclic oligosaccharides consisting of 6, 7, or 8 glucopyranose units (α-, β-, or γ-CD, respectively), well-known for their ability to complex and stabilize guest molecules, as well as for their solubilizing properties.^12^ Similar to other families of branched polymers,^13,14^ CD-branched polymers possess three-dimensional structure, improved multifunctionality, enhanced encapsulation capabilities, and high water solubility, but they offer several additional advantages.^15–17^ They include the opportunity of guest interaction with various binding sites within the 3D macromolecular network and the CD cavities, the constrained rotational freedom of the CD units due to the polymeric network and their mutual proximity, and the available hydroxyl groups on the CD rims, which enable conjugation of a supplementary component. These cross-linked CD polymers have shown great potential for both *in vitro* and *in vivo* applications,^15–17^ and their capability to encapsulate photoactivatable small molecules in a noncovalent manner and deliver them into tumor cells has been widely demonstrated.^18–20^ However, apart from branched CD polymers linking fluorescent tags as photoresponsive units,^21,22^ only very few examples of CD-branched polymers covalently integrating phototherapeutic functionalities are known to date. We have recently reported CD-branched polymers covalently integrating a nitric oxide (NO) photodonor (NOP) as a phototherapeutic component, without precluding the macromolecular network’s noncovalent ability to encapsulate additional hydrophobic guests.^23,24^ NO plays a multifaceted role in biology, not only as a key bioregulator of important physiological functions in the living body, including neurotransmission, vasodilation, and hormone secretion^25–29^ but also as a powerful “unconventional” antimicrobial and anticancer drug that is not subject to multidrug resistance (MDR).^30–34^ In this regard, the strict dependence of NO’s therapeutic activity, especially in cancer, on its concentration and site of action^35–37^ has made the light-controlled generation of this inorganic free radical highly appealing.^38–44^ In fact, the location and doses of NO released from NOPs can be finely regulated by the appropriate positioning of the excitation light in the specific area of interest and by the precise-tuning of the light intensity and irradiation time. However, it should be noted that once a NOP is introduced into a biological system, its photochemical performance can be significantly affected by specific interactions and side processes due to the complexity of the bioenvironment. Hence, monitoring the real NO released in cells is demanding and very challenging. To this end, the most commonly used approach is to add external probes to fluorescent assays.^45–50^ Nevertheless, it can lead to false positives or negatives due to unpredicted side reactions between the NOP and the NO probe, and competition between various biotargets and the fluorescent probe in the reaction with NO.^51^ An elegant strategy to overcome these drawbacks is based on the concept of “photorelease with fluorescent reporting.” ^52–54^ This approach is based on latent fluorogenic units as a part of the covalent skeleton of NOP, whose emission usually turns “on” after the NO release. In this way, the NO release process can be easily detected in real time, even in cells, by monitoring changes in the emission of the stable reporter generated in parallel with the liberated NO.^52–54^

We have previously designed and developed the bichromophoric dyad **1** (Scheme 1).^55^ It joins coumarin 120, a fluorophoric unit, and a nitroaniline-based NOP via an alkyl spacer. The compound has been designed so that the fluorophore’s typical blue emission is remarkably quenched by Förster Resonance Energy Transfer (FRET). Blue light excitation of **1** triggers NO release from the NOP unit and leads to the formation of the stable compound **2** in which the nitroaniline moiety is transformed into an aminophenol derivative. The latter is not a good energy acceptor, making the FRET process thermodynamically unfeasible and, therefore, restoring the fluorescence of the coumarin reporter.^55^

**Scheme 1:**
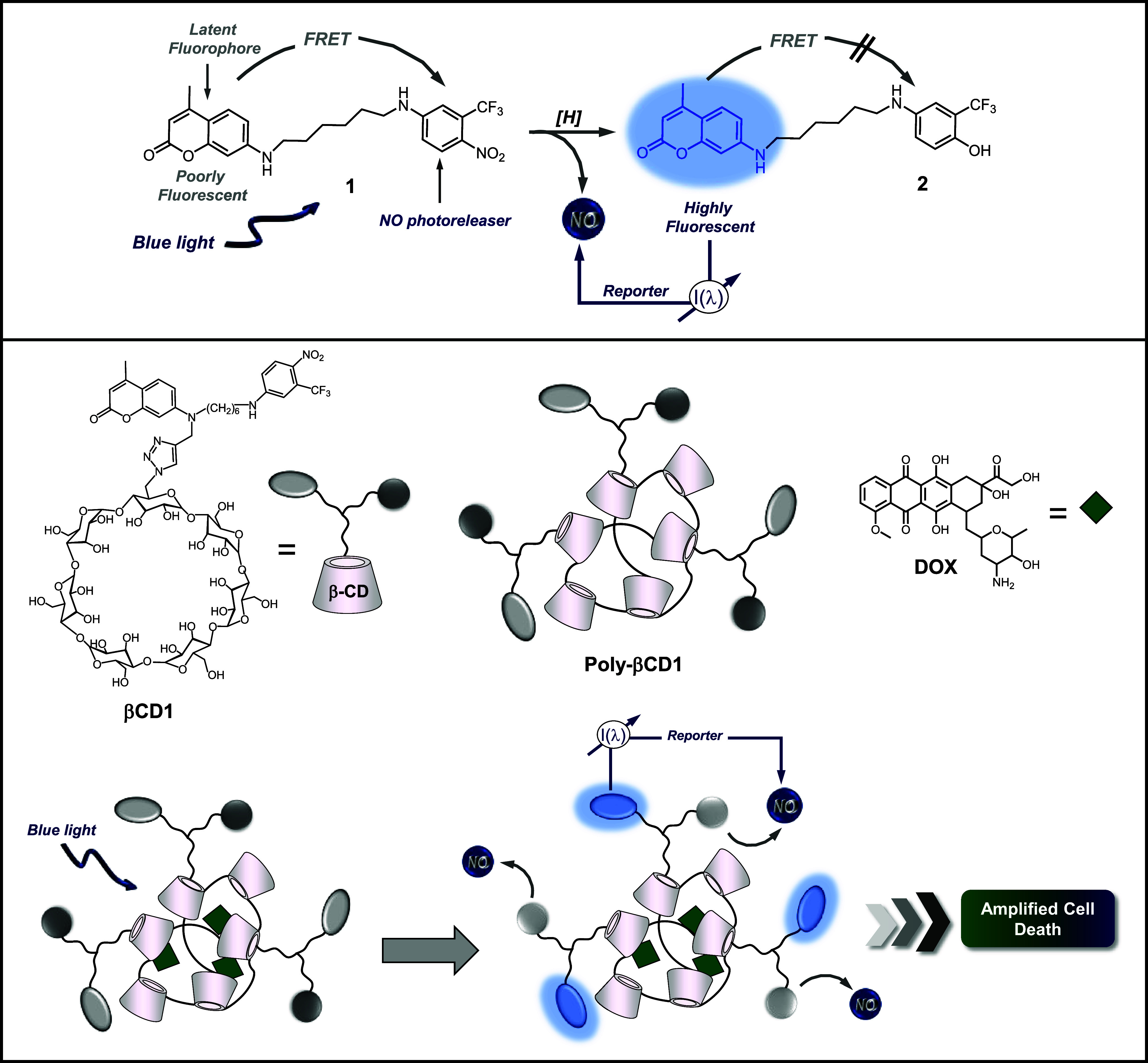
Molecular Structures and Working Principles of the Bichromophoric Dyad **1** and Its Stable Photoproduct **2** ^a^

a Molecular structures of the monomer **βCD1**, as a model compound, and **DOX**. Sketched structure of the branched polymer **Poly-βCD1**, its noncovalent assembly with **DOX** and its working principle.

The above scenario inspired us to develop a photoresponsive polymer capable of photoreleasing multiple NO molecules with fluorescent self-reporting in a confined region of space and, at the same time, encapsulating a conventional anticancer drug without precluding photochemical performance in view of multimodal photochemotherapeutic applications. To this end, we have devised the polymer **Poly-βCD1** (Scheme 1) in which the dyad **1** has been covalently integrated into a βCD-branched macromolecular scaffold.

**DOX** is a widely used anticancer drug belonging to the anthracycline family used as for treating a variety of tumors.^56^ However, MDR severely hinders the therapeutic efficacy of **DOX**. This resistance has been strictly related to increased efflux of this drug from tumor cells, consequent to the overexpression of ATP-binding cassette (ABC) transporters (efflux pumps).^57–59^ Moreover, the severe cardiotoxic side effects of this drug limit the cumulative tolerable dose in patients.^60^ In recent years, the combination of **DOX** with NO has emerged as a fascinating strategy to overcome MDR and to improve the overall antitumor activity. Besides acting directly as a cytotoxic agent, NO plays a key role in inhibiting the efflux pumps, mainly those responsible for MDR, thereby reducing the efflux of chemotherapeutic agents.^61–63^ In this regard, we have recently demonstrated that the combination of photoregulated NO release with **DOX** is a successful strategy to potentiate overall anticancer activity and minimize the therapeutic dose of the chemodrug.^64–67^ However, no systems combining **DOX** and NO photorelease with fluorescent reporting are known to date. On this regard, we report herein the synthesis, photochemical characterization, and biological validation of **Poly-βCD1** and its combination with **DOX**.

## Experimental Section

### Chemicals

6-Monoazido-6-monodeoxy-βCD (N_3_-βCD) was provided by Cyclolab Ltd. All chemicals were purchased from Sigma-Aldrich and used as received. All solvents used for the spectrophotometric studies were of spectrophotometric grade. Deionized ultrafiltered water was used throughout this study.

### Cell Lines

The human colorectal cancer cell lines HCT116 (CCL-247) and Caco-2 (HTB-37) were obtained from the American Type Culture Collection (ATCC, Manassas, VA, USA). HCT-116 cells were cultured in Roswell Park Memorial Institute medium (RPMI-1640), whereas Caco-2 cells were maintained in Dulbecco’s Modified Eagle Medium (DMEM). All media (Sigma-Aldrich, St. Louis, MO, USA) were supplemented with 2 mmol/L l-glutamine, 100 IU/mL penicillin, 100 μg/mL streptomycin, and 10% heat-inactivated fetal bovine serum (Sigma-Aldrich, St. Louis, MO, USA).

Cells were maintained at 37 °C in a humidified atmosphere containing 5% CO_2_ and were used within 15 passages after thawing. Mycoplasma contamination was routinely excluded by the PCR assay.

### Instrumentation

UV–vis absorption and fluorescence emission spectra were recorded with a Jasco spectrophotometer (mod. V-560) and a Spex Fluorolog-2 (mod. F-111) spectrofluorimeter, respectively, using 1 cm quartz cells. Fluorescence lifetimes were recorded with the same fluorometer equipped with a TCSPC Triple Illuminator. The samples were irradiated with a pulsed diode excitation source (Nanoled) at 455 nm. Ethanol was used to register the prompt at 455 nm. The system allowed for measurement of fluorescence lifetimes longer than 200 ps. The exponential fit of the fluorescence decay was obtained using eq 1

1 $$I\left(t\right)=\Sigma \alpha_{i}\exp{(}^{-t/\tau i})$$

Irradiation was performed in a thermostated quartz cell (1 cm path length, 3 mL capacity) under gentle stirring. A 100 mW continuum blue laser (λ_exc_ = 405 nm) having a beam diameter of ca. 1.5 mm was used as the light source.

Direct monitoring of NO release from solution samples was performed using an amperometric World Precision Instruments ISO-NO meter equipped with a data acquisition system, providing direct amperometric detection of NO with a short response time (<5 s) and a sensitivity range of 1 nM–20 μM. The analog signal was digitized with a four-channel recording system and transferred to a PC. The sensor was accurately calibrated by mixing standard solutions of NaNO_2_ with 0.1 M H_2_SO_4_ and 0.1 M KI according to the reaction 2

2 $$4H^{+}+2I^{-}+{2\mathrm{NO}}_{2}^{-}\to 2H_{2}O+2\mathrm{NO}+I_{2}$$

Irradiation was performed in a thermostated quartz cell (1 cm path length, 3 mL capacity) using the above continuum laser with λ_exc_ = 405 nm. NO measurements were carried out under stirring, with the electrode positioned outside the light path to avoid NO signal artifacts caused by photoelectric interference on the ISO-NO electrode.

Hydrodynamic diameter (*D*_H_), polydispersity index (PdI), and zeta potential (ζ) of the NPs were determined on a Zetasizer Nano ZS (Malvern Instruments Ltd., Malvern, U.K.).

Confocal fluorescence imaging was performed using a Leica TCS SP8 confocal laser scanning microscope (Leica Microsystems, Wetzlar, Germany) equipped with a 63× oil immersion objective (HC PL APO CS2, NA 1.4). The pinhole was adjusted to 1 Airy Unit. Images (1024 × 1024 pixels) were acquired at a scanning speed of 700 Hz with an eight-line averaging and a pixel size of 80 nm. Excitation was carried out at 405 nm, and emission signals were collected within the ranges of 415–520 and 530–650 nm. Bright field images were also recorded to assess the cellular morphology.

### Sample Preparation

**Poly-βCD1** was treated with EDTA, dialyzed for 24 h against Milli-Q water using a Spectra/Por regenerated cellulose membrane (Spectrum, Breda, The Netherlands; MWCO = 3.5 kDa), and subsequently lyophilized.

A stock solution of **DOX** (3.8 mM) was prepared in water. An appropriate aliquot was then added to a solution of **Poly-βCD1** (1 mg mL^–1^) to achieve the desired final **DOX** concentration, and the mixture was stirred at room temperature for 48 h.

### Photodecomposition Quantum Yields

Photodecomposition quantum yields (Φ_NO_) were determined at λ_exc_ = 405 nm within the 20% transformation of **βCD1** and **Poly-βCD1** by using eq 3

3 $$\Phi_{\mathrm{NO}}=\left[X\right]V/t\left(1-10^{-A}\right)I$$

where [*X*] is the concentration of phototransformed **βCD1** or **Poly-βCD1**, *V* is the volume of the irradiated sample, *t* is the irradiation time, *A* is the absorbance of the sample at the excitation wavelength, and *I* the intensity of the excitation light source. The concentrations of the phototransformed compounds were determined spectrophotometrically, taking into account the absorption changes at 380 nm and a Δε at this wavelength = 1 × 10^4^ M^–1^ cm^–1^,^68^ *I* was calculated by potassium ferrioxalate actinometry.

### Fluorescence Microscopy Imaging

For fluorescence imaging, Caco-2 cells were seeded onto poly-l-lysine-coated μ-Slide 8-well chambers (Ibidi, Germany) at a density of 2.0 × 10^5^ cells/mL and incubated for 24 h at 37 °C in 5% CO_2_. Cells were subsequently treated with **DOX** (1 μM), **Poly-βCD1** (1 mg mL^–1^), their combination, or PBS (control) for 4 h.

After treatment, the cells were washed with PBS and imaged. Fluorescence quantification was conducted by defining regions of interest (ROIs) corresponding to individual cells and calculating the mean fluorescence intensity within each ROI.

### Dark and Light-Induced Cytotoxicity

For experimental procedures, HCT116 cells (1.0 × 10^5^ cells/mL) and Caco-2 cells (1.5 × 10^5^ cells/mL) were seeded in 96-well plates (NUNC, Denmark) and allowed to adhere for 24 h. Cells were then treated with **DOX** (1 μM), **Poly-βCD1** (1 mg mL^–1^) or their combination for 4 h. Control cells received an equivalent volume of PBS. Following treatment, cells were washed with PBS and irradiated with a blue LED (415–420 nm; 10 mW cm^–2^). Fresh complete medium was added, and cells were further incubated for 48 h prior to analysis.

Cell viability was assessed using the MTT assay (3-(4,5-dimethylthiazol-2-yl)-2,5-diphenyl tetrazolium bromide; Sigma-Aldrich). After the treatments described above, an MTT solution (0.5 μg mL^–1^ final concentration) was added to each well, and the plate was incubated for 4 h to allow formazan crystal formation. The resulting insoluble formazan crystals were dissolved using an acidified isopropanol solution (0.04 N HCl). Absorbance was measured at 492 nm using a BioTek 800 TS microplate reader (Agilent Technologies, Santa Clara, CA, USA). Cell viability was expressed as a percentage relative to untreated control cells.

All experiments were performed in technical triplicate, and data are presented as the mean ± standard deviation (SD).

### Statistical Analysis

Statistical analyses were carried out using GraphPad Prism (GraphPad Software, San Diego, CA, USA).

Normality of the data distribution was assessed using the Shapiro–Wilk test, which confirmed that the samples followed a normal distribution; therefore, parametric statistical tests were applied. Single-parameter comparisons between two groups were performed using a two-tailed unpaired Student’s *t* test. Comparisons among three or more groups were conducted using one-way analysis of variance (ANOVA) followed by Tukey’s post hoc multiple comparison tests.

Statistical significance was defined as a *p*-value <0.05. Levels of significance were indicated as follows: *p* < 0.01 (*), *p* < 0.001 (**), and *p* < 0.0001 (*** or ###).

## Results and Discussion

### Design and Synthesis

To preserve the photochemical properties of molecular hybrid **1** upon integration into the CD polymer, we designed compound **1c** (Scheme 2 and SI), obtained via a two-step synthesis. It features a propargyl moiety linked to the amino group of coumarin 120 through a methylene spacer. This anchoring group was strategically selected to maintain the optimal distance between the NO photorelease and fluorescent reporting units, ensuring that both photoresponsive components operate as in **1** under light stimulation while permitting facile conjugation to either the azido-functionalized CD monomer (N_3_–CD) or the azido-functionalized CD polymer (poly-N_3_–CD) via CuAAC click chemistry. This approach, in which the complete bichromophoric dyad is assembled prior to conjugation with the CD unit, is considerably more advantageous than a stepwise functionalization strategy on the CD scaffold. In the latter case, the installation of the coumarin and nitroaniline components directly onto the CD would require prior protection of all free hydroxyl groups on the CD rim since these would otherwise participate in undesired side reactions during the synthetic steps needed to build the dyad. The subsequent deprotection and purification would add multiple low-yield steps, significantly reducing the overall synthetic efficiency. By contrast, CuAAC is fully orthogonal to the unprotected hydroxyl groups of the CD, allowing the preassembled dyad **1c** to be conjugated in a single, high-yielding, and regioselective step. Compound **1c** was then integrated in one step into both N_3_–CD and poly-N_3_–CD to give monomer **βCD1**, used as a model compound, and polymer **Poly-βCD1**, respectively (Scheme 2 and SI).

**Scheme 2:**
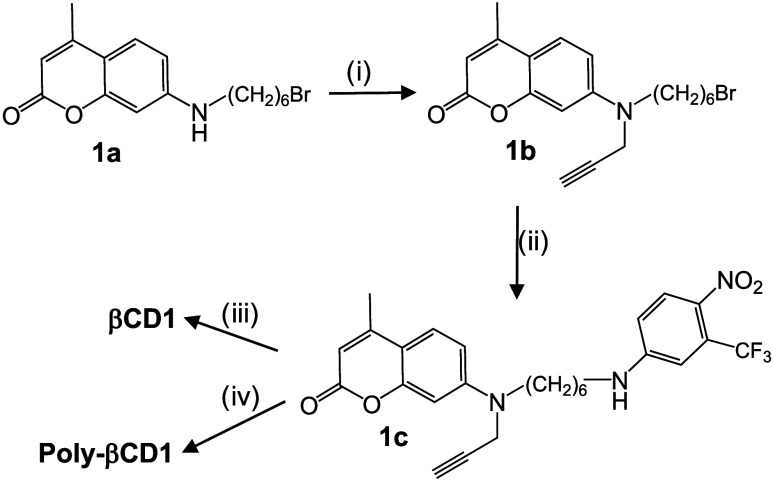
(i) K_2_CO_3_, Propargyl Bromide, Dry DMF, 60 °C, 3 Days; (ii) Cs_2_CO_3_, KI, TBAB, 4-Nitro-3-(trifluoromethyl)­aniline, CH_3_CN, 80 °C, Overnight; (iii) CuI, N_3_-βCD, DMF, 60 °C, Overnight; (iv) CuI, Poly-N_3_-βCD, H_2_O/DMF, 60 °C, Overnight

### Spectroscopic and Photochemical Behavior of βCD1 and Poly-βCD1

First, we explored the spectroscopic and photochemical behavior of the model compound **βCD1**, which is soluble in water up to *ca.* 2 mg mL^–1^. Its absorption spectrum shows a main band with a maximum at *ca.* 380 nm and extending up to *ca.* 500 nm (***a*** in Figure 1A). This spectral profile is very similar to that reported for the bichromophoric dyad **1** (see Scheme 1), and well reflects the arithmetic sum of the coumarin and nitroaniline components.^55^ This finding rules out (i) significant communication between the two chromophoric units in the ground state, even after their linking to the βCD scaffold, and (ii) potential self-inclusion of these chromophores within the βCD cavity. Visible light irradiation of the monomer **βCD1** induces a bleaching of the main absorption band (Figure 1A) accompanied by a slight shift to the blue. These changes of the spectral profile are identical to those observed for the molecular hybrid **1**,^55^ consistent with the NO photorelease from the nitrogroup of the nitroaniline unit and its evolution to a phenol derivative after H-transfer (see Scheme 1), in line with the photochemical pathway previously proposed in the case of the isolated NOP.^68^ NO photorelease was directly confirmed by its amperometric detection by using an NO ultrasensitive electrode. Figure 1B unambiguously shows that the NO release is exclusively regulated by the presence of blue light. The quantum yield for the NO photogeneration was Φ_NO_ = (5 ± 0.5) × 10^–4^. This value is comparable to that already reported for the water-soluble individual NOP unit,^68^ confirming that the βCD ring does not affect the excited-state behavior of the NO photoreleasing component. **βCD1** exhibits a low fluorescence emission (***a*** in Figure 1C). This result confirms that the FRET quenching mechanism, operating in the dyad **1**, also occurs after its integration in the CD scaffold, supporting the validity of our synthetic design. Accordingly, photoexcitation of **1** leads to a substantial revival of the typical emission of the coumarin fluorophore as a result of the release of NO and suppression of the FRET process. These results prove that bichromophoric dyad **1** preserves its photochemical properties well after its grafting into the βCD scaffold.

**Figure 1:**
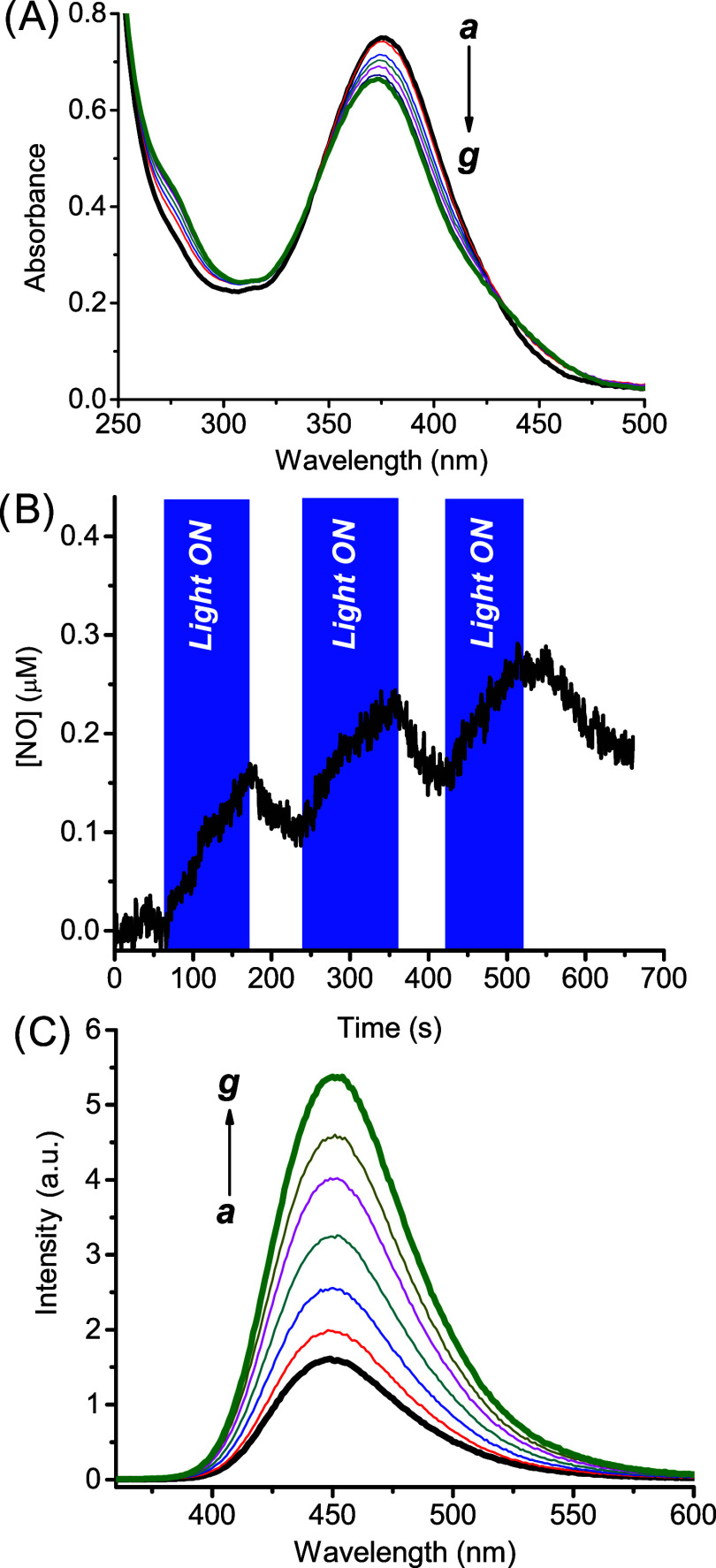
(A) Absorption spectral changes observed upon irradiation with λ_exc_ = 405 nm at regular time intervals of 5 min from 0 min (***a***) to 30 min (***g***) of an aqueous solution of **βCD1** (30 μM). (B) NO release profile observed upon alternate cycle of irradiation with λ_exc_ = 405 nm of an aqueous solution of **βCD1** (30 μM). (C) Evolution of the fluorescence emission spectra corresponding to the sample of (A), and recorded at λ_exc_ = 350 nm. *T* = 25 °C.

**Poly-βCD1** exhibits a water dispersibility of more than 10 mg mL^–1^, which is *ca.* 5-fold higher than that of the monomeric **βCD1**, as a result of the presence of multiple βCD units. Its absorption spectrum (***a*** in Figure 2A) is basically identical to that of the monomer, confirming that, even in this case, both chromophores do not interact with the βCD cavity. A set of photolysis experiments was then carried out with **Poly-βCD1** and the results are summarized in Figure 2.

**Figure 2:**
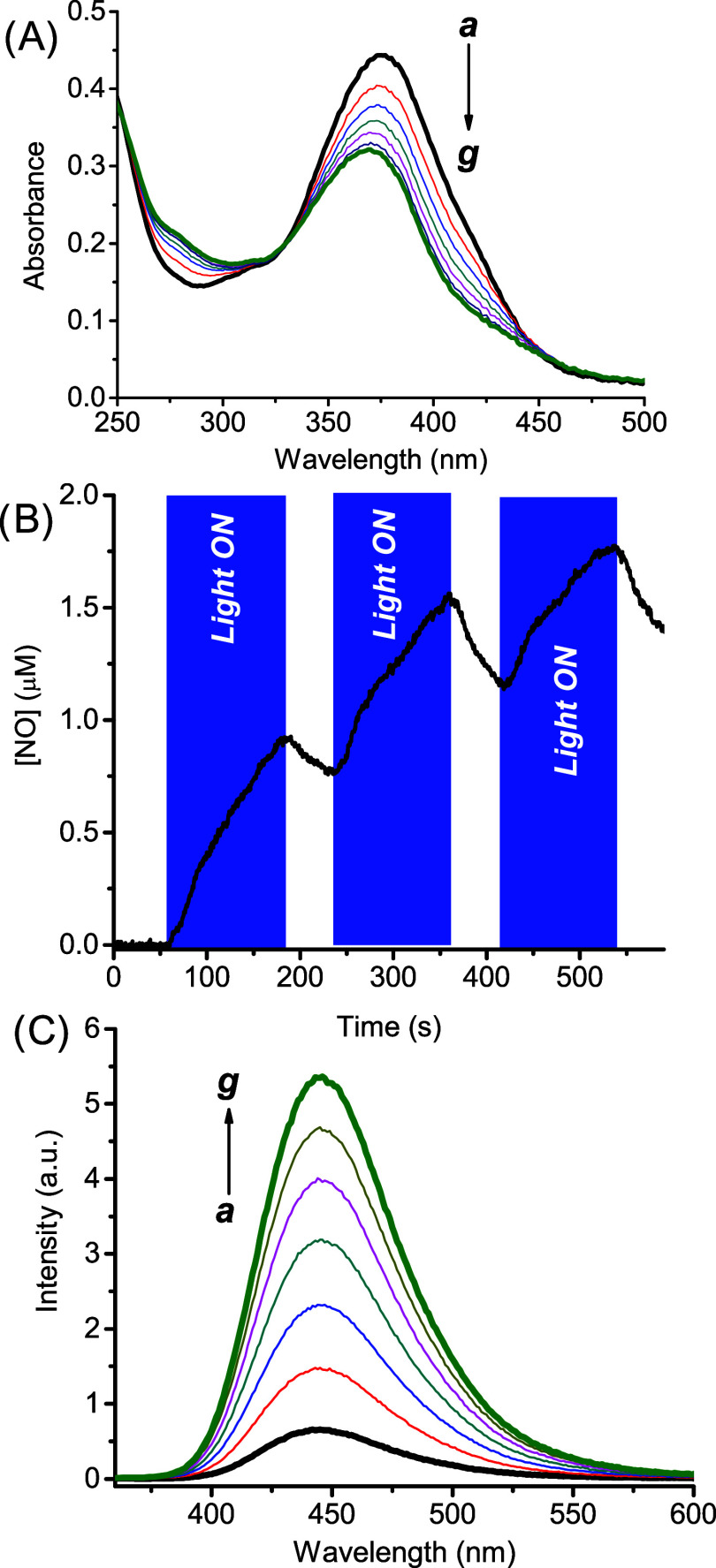
(A) Absorption spectral changes observed upon irradiation with λ_exc_ = 405 nm at regular time intervals of 5 min from 0 min (***a***) to 30 min (***g***) of an aqueous suspension of **Poly-βCD1** (0.5 mg mL^–1^). (B) NO release profile observed upon alternate cycle of irradiation with λ_exc_ = 405 nm of an aqueous suspension of **Poly-βCD1** (0.5 mg mL^–1^). (C) Evolution of the fluorescence emission spectra corresponding to the sample of (A), and recorded at λ_exc_ = 350 nm. *T* = 25 °C.

The experimental data confirm the same photobehavior observed for the monomer, such as (i) bleaching of the main absorption band accompanied by a slight blue-shift, (ii) photoregulated release of NO, and (iii) significant revival of the fluorescence of the coumarin unit. Interestingly, the quantum yield for the NO photogeneration was Φ_NO_ = (4.0 ± 0.3) × 10^–3^, a value almost 1 order of magnitude larger than that observed for the **βCD1**. This result accords well with the values recently observed for NOPs using the same chromogenic nitroaniline derivative unit, either linked to similar CD-branched polymers,^23,24^ or entrapped in polymeric micelles.^69^ Such enhanced photoreactivity is not uncommon in radical-mediated reactions and is ascribed to the active role of the polymer network as a reactant in providing abstractable H atoms close to the phenoxy-radical intermediate involved in the mechanism of NO photorelease.^68^

### Spectroscopic and Photochemical Behavior of the Poly-βCD1/DOX Complex

CD-based branched polymers have proven to be suitable carriers for several chemotherapeutics,^20,24^ including **DOX**.^70,71^ Figure 3A shows the absorption features of aqueous solutions of the mixture **Poly-βCD1/DOX**, spectrum ***a***, the individual **Poly-βCD1** and **DOX**, spectra ***b*** and ***c***, respectively, and their mathematic sum (spectrum ***d***). The nonperfect overlap between spectra ***a*** and ***d*** suggests the occurrence of some specific interactions between the two components in the ground state. Entrapment of **DOX** into the polymer was also confirmed by dynamic light scattering (DLS) measurements. In an aqueous medium, **Poly-βCD1** forms a nanoassembly with an average hydrodynamic diameter (*D*_H_) = 280 ± 4 nm and a polydispersity index (PDI) = 0.37 (***a*** in the Inset Figure 3A). In the presence of **DOX**, we observed a slight increase in D_H_ up to 301 ± 5 nm and a reduction in PDI to 0.30 (***b*** in the Inset Figure 3A).

**Figure 3:**
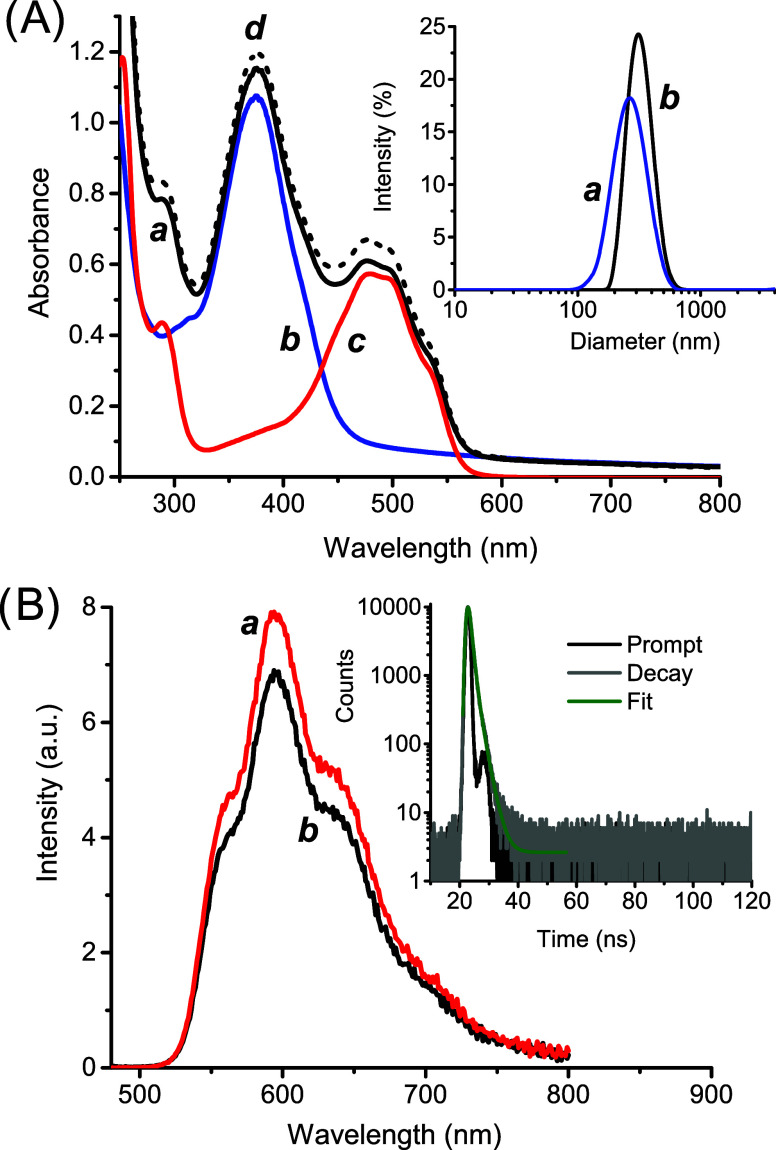
(A) Absorption spectra of an aqueous suspension of **Poly-βCD1** (***a***) in the presence and (***b***) in the absence of **DOX**, (***c***) an aqueous solution of **DOX**, and (***d***) the arithmetic sum of spectra (***b***) and (***c***). The inset shows the hydrodynamic diameter of an aqueous suspension of **Poly-βCD1** in the absence (***a***) and in the presence (***b***) of **DOX**. (B) Fluorescence emission spectra (λ_exc_ = 470 nm) of **DOX** in the absence (***a***) and in the presence (***b***) of **Poly-βCD1**. The inset shows the fluorescence decay and the related biexponential fitting monitored at λ_em_ = 590 nm. [**Poly-βCD1**] = 1 mg mL^–1^; [**DOX**] = 50 μM; *T* = 25 °C.

Steady-state and time-resolved fluorescence measurements further corroborated the formation of the noncovalent **Poly-βCD1**/**DOX** complex. In aqueous medium, **DOX** exhibits the typical structured emission with a maximum at 590 nm (spectrum ***a*** in Figure 3B). According to the literature, the related fluorescence decay is essentially monoexponential with a lifetime of 1.01 ns.^71^ The addition of **Poly-βCD1** results in slight changes in fluorescence intensity with no change in the spectral shape (spectrum ***b*** in Figure 3B). In contrast, the decay kinetics were modified. In this case, biexponential decay analysis applies fairly well, giving a faster component with τ_1_ = 0.87 ns and related amplitude α_1_ = 78% and a second, slower component, with τ_2_ = 1.97 ns and related amplitude α_2_ = 22% (inset Figure 3B). These changes in the fluorescence dynamics reflect quite well what has already been observed for **DOX** encapsulated in other CD-branched polymers^71^ and may reflect the localization of **DOX** in different nanoenvironments of the polymer. The **Poly-βCD1**/**DOX** complex was stable for more than one month, as confirmed by the negligible changes of its absorption and emission spectra and the DLS analysis.

One of the main prerequisites for photoactivatable therapeutics, when combined with chemotherapeutics, is the preservation of their photochemical performances. In general, this is not trivial result, since the photoresponse may be modified or even precluded by undesired bimolecular reactions (i.e., photoinduced energy/electron transfer) between the two components, especially when they are confined in their proximity. Figure 4A,B shows the absorption and fluorescence emission (this latter in the region of the coumarin fluorophore) spectral changes observed upon blue light irradiation of the **Poly-βCD1**/**DOX** nanoassembly. The spectral profiles match quite well those already observed in the absence of **DOX** and characterized by the bleaching and blue-shift of the absorption band at 380 nm and the dramatic revival of the typical emission of the coumarin fluorophore with a maximum at 450 nm (see Figure 2A,C). Interestingly, under these experimental conditions, we did not observe any significant photodegradation of **DOX**, as confirmed by its unaltered fluorescence emission after the photolysis (Figure 4C). The photobehavior observed is in excellent agreement with the release of NO, which was again supported by its direct amperometric detection (Figure 4D). Interestingly, the NO photogeneration quantum yield was Φ_NO_ = (3.9 ± 0.3) × 10^–3^ that is a value similar to that observed in the absence of **DOX** (*vide supra*). Therefore, these findings clearly suggest that photoactivation of the **Poly-βCD1**/**DOX** complex results in excellent preservation of the NO photorelease and the polymer’s fluorescent reporting properties, while conserving the chemical integrity of the embedded chemodrug, as sketched in Scheme 1.

**Figure 4:**
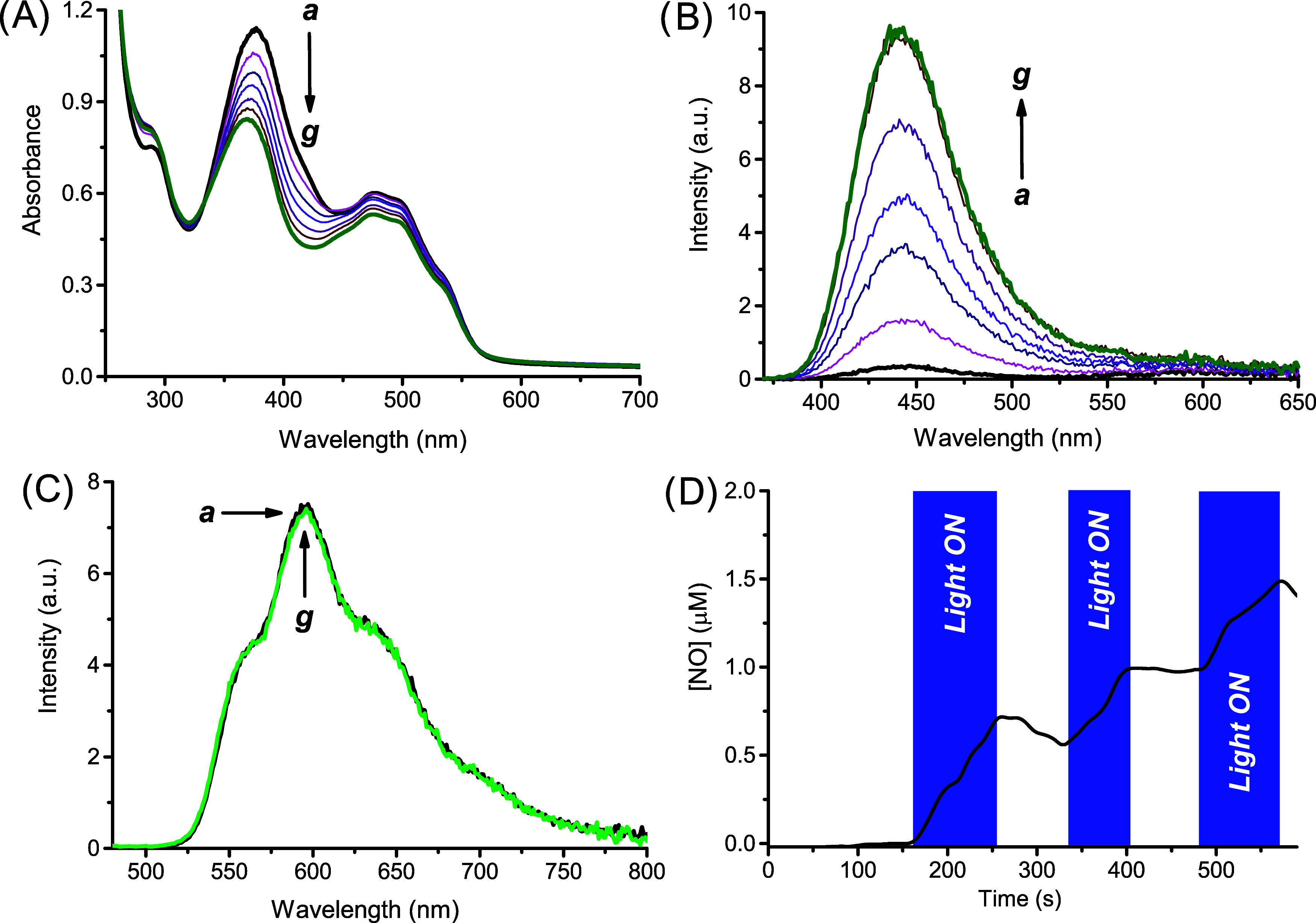
(A) Absorption spectral changes observed upon irradiation with λ_exc_ = 405 nm at regular time intervals of 5 min from 0 min (***a***) to 30 min (***g***) of an aqueous suspension of **Poly-βCD1** in the presence of **DOX**. (B) Evolution of the fluorescence emission spectra corresponding to the sample of (A), and recorded at λ_exc_ = 350 nm. (C) Fluorescence emission spectra related to the same sample recorded at λ_exc_ = 470 nm and acquired after 0 min (***a***) and 30 min (***g***) of irradiation with λ_exc_ = 405 nm. (D) NO release profile observed upon alternate cycle of irradiation with λ_exc_ = 405 nm of an aqueous suspension of **Poly-βCD1** in the presence of **DOX**. [**Poly-βCD1**] = 1 mg mL^–1^; [**DOX**] = 50 μM; *T* = 25 °C.

### Cell Imaging and Viability Studies

To validate the suitability of **Poly-βCD1** and its complex with **DOX** as an NO photoreleaser with fluorescent reporting in a cell environment, we performed confocal fluorescence microscopy experiments with **DOX**-resistant Caco-2 cancer cells. A solution of **Poly-βCD1** was incubated with this cell line for 4 h and subsequently irradiated with 405 nm light for different times. Figure 5A (panels a–d) shows the fluorescence images taken before and immediately after each step of irradiation. It can be seen that the negligible emission of the non irradiated sample evolves into the appearance of the distinctive blue emission of the coumarin reporter, whose intensity increases as a function of the irradiation time. These findings perfectly match the photobehavior observed in solution, confirming that the working principle of the bichromophoric dyad grafted onto the polymer also operates well in the cell environment. A close examination of the fluorescence microscopy images suggests a localization of the polymer mainly at the cytoplasmic level.

**Figure 5:**
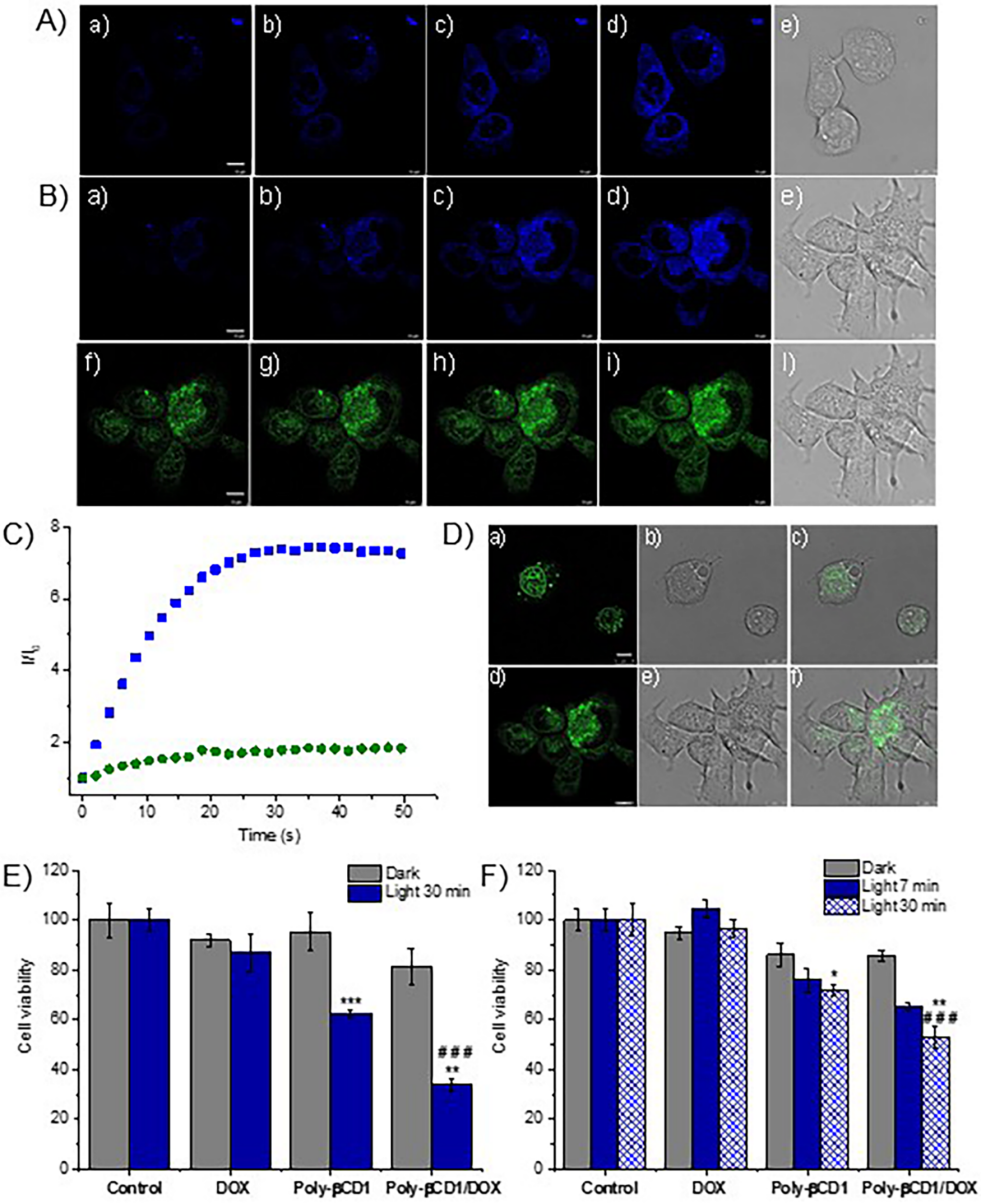
(A) Confocal laser fluorescence microscopy images of Caco2 cancer cells incubated with **Poly-βCD1** for 4 h and taken after 0 s (a), 3 s (b), 9 s (c), and 49 s (d) of irradiation with blue laser at λ_exc_ = 405 nm and collecting fluorescence in the blue channel (415–520 nm); panel (e) shows the confocal microscopy bright field image. (B) Confocal laser fluorescence microscopy images of Caco2 cancer cells incubated with **Poly-βCD1**/**DOX** for 4 h and taken after 0 s (a, f), 3 s (b, g), 9 s (c, h), and 49 s (d, i) of irradiation with blue laser at λ_exc_ = 405 nm and collecting fluorescence in the blue channel (415–520 nm) (panels a–d) and in the green channel (530–650 nm) (panels f–i); panels (e, l) show the confocal microscopy bright field images. (C) Evolution of the mean fluorescence intensity monitored in the blue (■) and the green (●) channel in Caco2 cancer cells incubated with **Poly-βCD1**/**DOX**. (D) Confocal laser fluorescence microscopy images of Caco2 cancer cells incubated with **DOX** (a) or **Poly-βCD1**/[**DOX**] (d) for 4 h, taken at λ_exc_ = 488 nm and collecting fluorescence in the green channel (530–650 nm); panels (b, e) show the related confocal microscopy bright field images whereas panels (c, f) show the related overlay images. [**PolyCNO**] = 1 mg mL^–1^; [**DOX**] = 1 μM. Scale bar = 10 μm. Cell viability of HCT-116 (E) and Caco2 (F) cancer cells incubated with **DOX**, **Poly-βCD1** or their combination and either kept in the dark or irradiated with blue light (λ_exc_ = 415–420 nm; 10 mW cm^–2^). All experiments were carried out with [**Poly-βCD1**] = 1 mg mL^–1^ and [**DOX**] = 1 μM. Data are expressed as mean percentage ± SD of three independent experiments, carried out in triplicate. *p < 0.01 vs dark; **p < 0.001 vs dark; ***p < 0.0001 vs dark; ###*p* < 0.0001 vs **Poly-βCD1** under light conditions.

Analogous experiments were also performed with the **Poly-βCD1**/**DOX** complex. In this case, the fluorescence emission was monitored in both the blue and green channels to detect the coumarin reporter and **DOX**, respectively. As shown in Figure 5B, we observed a remarkable intensification of the fluorescence in the blue channel (panels a–d) and only a slight increase of the emission intensity in the green channel (panels f–i) upon illumination (see also Figure 5C). This behavior parallels that observed for the **Poly-βCD1**/**DOX** complex in solution, confirming the activation of the NO reporter as a consequence of the NO release, also in the presence of **DOX**, and the preservation of the chemical integrity of the chemodrug. Fluorescence images of the sample containing **DOX** alone were then acquired before initiating the irradiation cycle and compared with those of sample **Poly-βCD1**/**DOX**. As expected, **DOX** is mainly localized in the nucleus (Figure 5D, panels a–c). On the other hand, when the drug is incubated with **Poly-βCD1**, partial colocalization in the cytoplasm is observed (Figure 5D, panels d–f). This finding agrees well with the entanglement of **DOX** into the polymeric network of **Poly-βCD1**, suggesting that negligible displacement occurs in the cell environment during the 4h of incubation. At this regard, since no changes in the sizes of the nanoassembly were noted after irradiation, we believe that, according to similar cases reported in the literature,^70,72^ the release mechanism of **DOX** from the polymer is more likely mainly driven by simple noncovalent exchange. The capability of **Poly-βCD1** to deliver NO alone and in combination with **DOX**, prompted us to perform some preliminary cell viability experiments to evaluate the therapeutic action of the polymer and its complex with the chemodrug, in the dark and under light irradiation and, for comparison with **DOX** alone under the same experimental conditions. To this end, we carried out experiments with both HCT-116 **DOX**-sensitive and Caco-2 **DOX**-resistant cancer cells. Given the well-known side effects of **DOX** limiting its cumulative tolerable dose in patients,^60^ for these experiments, we have chosen a **DOX** concentration of 1 μM, which is 5-fold lower than the usual therapeutic concentration.

The results obtained with HCT-116 cells (Figure 5E) show a negligible effect of **DOX** on cell viability in the dark, in accordance with the undertherapeutic doses used, and only a slight increase under irradiation. This latter is more likely due to **DOX**’s known slight photodynamic action, which produces reactive oxygen species under light excitation.^73^ **Poly-βCD1** was nontoxic in the dark but induced significantly reduction of cell viability under photoexcitation, more likely due to the cytotoxic effect of the photogenerated NO. Interestingly, the combination **Poly-βCD1**/**DOX** exhibited low dark toxicity but a significant potentiated effect under light irradiation if compared with **Poly-βCD1** alone. **DOX** did not affect at all the cell viability of Caco-2 cancer cells, neither in the dark nor under light irradiation (Figure 5F), consistent with their **DOX**-resistant nature. **Poly-βCD1** was nontoxic in the dark but led to a reduction in cell viability under light. Similar to the results observed for **DOX**-sensitive cells, also in the case of Caco-2 cancer cells, the combination **Poly-βCD1**/**DOX** induces a high level of mortality under irradiation if compared with the polymer alone, clearly depending on the irradiation time. Note that the amplified reduction in viability observed with both cell lines in the case of the **Poly-βCD1**/**DOX** samples cannot be trivially ascribed to an additive effect of the two components.

Furthermore, the very similar values for the NO photogeneration quantum yields of **Poly-βCD1** in the absence and in the presence of **DOX** (*vide supra*) rule out that the larger decrease of the cell viability observed for the combination of the **Poly-βCD1**/**DOX** complex is due to a higher concentration of NO photogenerated if compared with the free **Poly-βCD1**. Rather, these findings suggest the occurrence of a key role of the NO photogenerated in enhancing the **DOX** therapeutic effect.

## Conclusions

We have reported an intriguing example of phototherapeutic CD-based branched polymer covalently integrating tailored functionalities for the photoregulated NO release with fluorescent reporting. This polymer is highly dispersible in aqueous medium and generates the NO radical simultaneously with a fluorescent reporting function, with a quantum yield almost 1 order of magnitude larger than that observed for its monomer analogue. The polymeric construct can entrap the anticancer **DOX**, preserving its photochemical properties while maintaining the chemical integrity of the chemodrug. Both the free polymer and its **DOX** complex internalize into cancer cells, where photogeneration of NO occurs as in solution and can be simply visualized in real time by fluorescence microscopy with the aid of the fluorescent reporter. **Poly-βCD1** is tolerated by cancer cells in the dark but induces cell death under light irradiation, most likely due to the cytotoxic action of NO. Toxicity experiments carried out with **DOX**-sensitive and **DOX**-resistant cancer cells at a **DOX** concentration well below the therapeutic value showed an amplified cell death when combined with **Poly-βCD1**, pointing to a key action of NO in enhancing the therapeutic effect of **DOX**. Given the multiple roles of NO as a cytotoxic agent and as an inhibitor of the ABC transporters, mainly responsible for **DOX** cell efflux, detailed biological studies aimed at shedding light on the mechanism underlying this NO-induced enhancement of **DOX** activity are already ongoing in our laboratories. *In vivo* studies are also planned to evaluate the translatability of the *in vitro* photochemotherapeutic effects demonstrated herein.

In view of the very critical dependence of the biological effects of NO by its concentration and location, the present photoactivatable polymer can represent an intriguing photopharmacological weapon for the delivery of multiple NO molecules in a confined region of space with instantaneous visualization without the addition of external probes. Furthermore, given the increasing interest in combining NO with chemotherapeutics, these findings may offer promising prospects for enhancing the antitumor efficacy of **DOX** while simultaneously mitigating its adverse effects, particularly within low-dosage.

## Supplementary Material



## Notes

Details of synthetic procedures; NMR and mass spectra (PDF)
