# Supplementary material for: Phototherapeutic β‑Cyclodextrin-Branched Polymer Releasing Nitric Oxide with Fluorescent Self-Reporting and Its Combination with Doxorubicin
Source: Biomacromolecules. 2026 Apr 15;27(5):3399–409. doi: 10.1021/acs.biomac.6c00462 (PMC13169392; doi:10.1021/acs.biomac.6c00462)
Supplement: Supplementary file 1 [file bm6c00462_si_001.pdf]

Supporting Information

A Phototherapeutic β-Cyclodextrin Branched Polymer Releasing Nitric Oxide with Fluorescent Self-Reporting and its Combination with Doxorubicin

Marta Perez-Lloret,[†,#] Cristina Parisi,[†,#] Francesca Laneri,[†] Gian Marco Leone,[‡] Cristina Barbagallo,[‡] Katia Mangano,[‡] Ferdinando Nicoletti,[‡] Szabolcs Béni,[¥] Milo Malanga,[§] and Salvatore Sortino[†,]*

[†]*PhotoChemLab, Department of Drug and Health Sciences, University of Catania, I-95125 Catania, Italy.*

[‡]*Department of Biomedical and Biotechnological Sciences, University of Catania, I-95125 Catania, Italy.*

[¥]*Department of Analytical Chemistry, Institute of Chemistry, H-1117 Budapest, Hungary.*

[§]*CarboHyde Ltd., Berlini u. 47-49, H-1045, Budapest, Hungary*

*Email: ssortino@unict.it

[#]These authors equally contributed to this work.

Synthesis and characterization

Compounds **1a** and **poly-N$_3$-βCD** was synthetized according to our previously reported procedure.[1S,2S]

7-((6-bromohexyl)(prop-2-yn-1-yl)amino)-4-methyl-2H-chromen-2-one (1b). Compound **1a** (200 mg, 0,6 mmol) and K_2CO_3 (82 mg, 0,6 mmol) were suspended in dry DMF (5 mL) under inert atmosphere. Propargyl bromide (6,0 mmol) (purity *ca.* 70%) was diluted with DMF (1 mL) and added dropwise for 10 minutes to the suspension. The mixture was heated at 60 ºC for 3 days, then poured into water (100 mL) and extracted with dichloromethane (3 x 30 mL). The organic phases were combined and washed with water (5 x 50 mL), dried over Na_2SO_4, and finally concentrated under vacuum. The obtained solid was purified by chromatographic column (EtOAc/cyclohexane 1:3) to afford **1b** as a yellowish powder (136 mg, 60% yield) Rf = 0,77 (EtOAc/cyclohexane 1:1). ^{1}H NMR (600 MHz, $CDCl_3$) δ 7.43 (d, J = 8.9 Hz, 1H, C**H**Ar), 6.70 (dd, J = 8.9, 2.6 Hz, 1H, C**H**Ar), 6.63 (d, J = 2.7 Hz, 1H, C**H**Ar), 6.00 (d, J ≈ 1.0–1.2 Hz, 1H, C**H**Ar), 4.05 (d, J = 2.4 Hz, 2H, C**H$_2$**C≡CH), 3.42 (m, 4H, NC**H$_2$**CH$_2$CH$_2$CH$_2$CH$_2$C**H$_2$**Br), 2.34 (d, J ≈ 1.0–1.2 Hz, 3H, Ar-C**H$_3$**), 2.24 (t, J = 2.4 Hz, 1H, CH$_2$C≡C**H**), 1.91 – 1.83 (quint, J = 7.7 Hz, 2H, NCH$_2$CH$_2$CH$_2$CH$_2$C**H$_2$**CH$_2$Br), 1.69 (quint, J = 7.7 Hz, 2H, NCH$_2$C**H$_2$**CH$_2$CH$_2$CH$_2$CH$_2$Br), 1.53 – 1.45 (quint, J = 7.7 Hz 2H, NCH$_2$CH$_2$CH$_2$C**H$_2$**CH$_2$CH$_2$Br), 1.38 (quint, J = 7.7 Hz, 2H, NCH$_2$CH$_2$C**H$_2$**CH$_2$CH$_2$CH$_2$Br). ^{13}C NMR (151 MHz, $CDCl_3$) δ 161.99, 155.67, 152.86, 150.59, 125.58, 110.65, 110.04, 109.63, 99.55, 79.10, 72.57, 51.71, 40.50, 33.83, 32.71, 28.03, 27.22, 26.30, 18.57. ESI-MS: m/z found: 376.09 [M+H], calculated for $C_{19}H_{22}BrNO_2$ 376.09.

4-methyl-7-((6-((4-nitro-3-(trifluoromethyl)phenyl)amino)hexyl)(prop-2-yn-1-yl)amino)-2H-chromen-2-one (1c). Compound **1b** (100 mg, 0,27 mmol), 4-nitro-3-(trifluoromethyl)aniline (110 mg, 0.53 mmol), Cs_2CO_3 (86 mg, 0,27 mmol), KI (44 mg, 0,27 mmol) and tetrabutylammonium bromide (TBAB, 10 mg, 0,03 mmol) were added in sequence to vigorously stirred acetonitrile (10 mL) and the suspension was refluxed overnight. After cooling, the mixture was filtered to remove solid residue, the filtrate was concentrated under vacuum and the obtained solid was purified by column chromatography (DCM/EtOAc 99:1) to yield **1c** as a yellow viscous oil (67 mg, 50% yield), Rf = 0,20. ^{1}H NMR (600 MHz, $CDCl_3$) δ 8.01 (d, J = 8.8 Hz, 1H, C**H**Ar), 7.44 (d, J = 8.8 Hz, 1H, C**H**Ar), 6.88 (s, 1H, C**H**Ar), 6.72 (d, J = 8.8 Hz, 1H, C**H**Ar), 6.65 (m, 2H, C**H**Ar), 6.00 (s, 1H, C**H**Ar), 4.77 (bs, 1H, N**H**), 4.09 (s, 2H, C**H$_2$**C≡CH), 3.44 (t, J = 7.6 Hz, 2H, NC**H$_2$**CH$_2$CH$_2$CH$_2$CH$_2$CH$_2$NH), 3.24 (t, J = 7.0 Hz, 2H, NCH$_2$CH$_2$CH$_2$CH$_2$CH$_2$C**H$_2$**NH), 2.36

(s, 3H, Ar-C**H₃**), 2.24 (t, 1H, $CH_2C≡C$**H**), 1.71 (m, J = 14.8, 7.4 Hz, 4H, NCH_2C**H₂**CH_2CH_2C**H₂**CH_2NH), 1.49- 1.43 (m, J = 13.6, 6.9 Hz, 4H, NCH_2CH_2C**H₂**C**H₂**CH_2CH_2NH). ^{13}C NMR (151 MHz, $CDCl_3$) δ 161.90, 155.63, 152.88, 151.93, 150.08, 136.59, 129.34, 125.74, 123.32, 121.51, 112.59, 111.22, 110.53, 110.16, 100.22, 78.74, 73.07, 51.91, 43.45, 41.04, 29.85, 29.01, 27.06, 26.84, 26.78, 18.63. ESI-MS: m/z found: 502.19 [M+H], calculated for $C_{26}H_{26}F_3N_3O_4$ 502.19.

βCD1. Compound **1c** (3.2 mg, 6.4 μmol) was dissolved in DMF (800 μL). Solid N_3-βCD (4.9 mg, 4.3 μmol) was added to the solution, followed by water (200 μL) to give a final H_2O:DMF ratio of 1:4 (v/v). CuI (1.2 mg, 6.4 μmol) was then added under vigorous stirring, and the mixture was heated at 60 °C overnight. The mixture was filtered, the filtrate was concentrated under vacuum and the residue was precipitated with acetone (5 mL). The solid obtained by filtration was purified by silica gel column ($ACN/H_2O/NH_3$ 10:5:2) to yield a yellow solid in a quantitative yield. ^{1}H NMR (600 MHz, MeOD) δ (ppm): 8.02 (d, 1H, C**H**Ar), 7.55 (d, 1H, C**H**Ar), 6.99 (s, 1H, C**H**Ar), 6.90 (dd, 1H, C**H**Ar), 6.76 (dd, 1H, C**H**Ar), 6.68 (s, 1H, C**H**Ar), 5.97 (d, 1H, C**H**Ar), 5.18 (d, 1H, CD-**H**1), 4.99-4.92 (7H, CD-**H**1, CD-**H**6a), 3.95-3.40 (42H, m, H2, H3, H4, H5, H6, H6b), 3.61 (q, 2H, – $(C\mathbf{H_2})_6$–), 3.22 (t, 2H, – $(C\mathbf{H_2})_6$–), 2.45 (d, 3H, C**H**₃-Ar), 1.75 – 1.66 (m, 4H, – $(C\mathbf{H_2})_6$–), 1.54 – 1.43 (m, 4H, – $(C\mathbf{H_2})_6$–). Carbon assignments based on HSQC-DEPT-edited in MeOD (151 MHz) δ (ppm): 130.61 (**C**HAr), 126.99 (**C**HAr), 112.63 (**C**HAr), 111.45 (**C**HAr), 111.11 (**C**HAr), 109.26 (**C**HAr), 99.73 (**C**HAr), 104.12 (**C**Trz), 103.86 (**C1**), 83.02 (**C4**), 70.81 (**C2**), 74.29-73.61 (**C3/C5**), 61.89 (**C6**), 18.52 (**C**H_3-Ar). ESI-MS: m/z found: 1661.50 [M+H], calcd for $C_{68}H_{95}F_3N_6O_{38}$ 1661.57.

Poly-βCD1. 135 mg of ***poly-N₃-βCD*** (MW 122 ± 3 kD) integrating 10 μmol of azido groups, was dissolved in water (350 μL). A DMF-solution (1.4 mL) of **1c** (7,7 mg, 15 μmol), and CuI (4,4 mg, 15 μmol) were sequentially added. The mixture was heated at 60 ºC under vigorous stirring and reacted overnight. After cooling, the reaction mixture was filtered and the mother liquor was extracted with dichloromethane (3 x 10 mL). The aqueous phase was dialyzed against deionized water (regenerated cellulose, MWCO 12 kDa) for 24 h (water changed every 3 h) and the solvent evaporated until dryness under vacuum to yield a yellow powder. The typical absorption spectrum of the bichromophoric dyad after dialysis, unambiguously confirmed the success of the click reaction. Based on the absorbance value at 380 nm, the amount of **1c** linked to the polymer was estimated to be $7.4x10^{-3}$ μM mg^{-1}, which indicates a quantitative transformation of ***poly-N₃-βCD*** into ***Poly-βCD1.***

References

1S. Marino, N.; Perez-Lloret, M.; Blanco, A. R.; Venuta, A.; Quaglia, F.; Sortino, S. Photoantimicrobial polymeric films releasing nitric oxide with fluorescence reporting under visible light. *J. Mater. Chem. B* **2016**, *4*, 5138-5143.

2S. Malanga, M.; Bálint, M.; Puskás, I.; Tuza, K.; Sohajda, T.; Jicsinszky, L.; Szente, L.; Fenyvesi, E. Synthetic strategies for the fluorescent labeling of epichlorohydrin-branched cyclodextrin polymers. *Beilstein J Org Chem.* **2014**, *10*, 3007–3018.

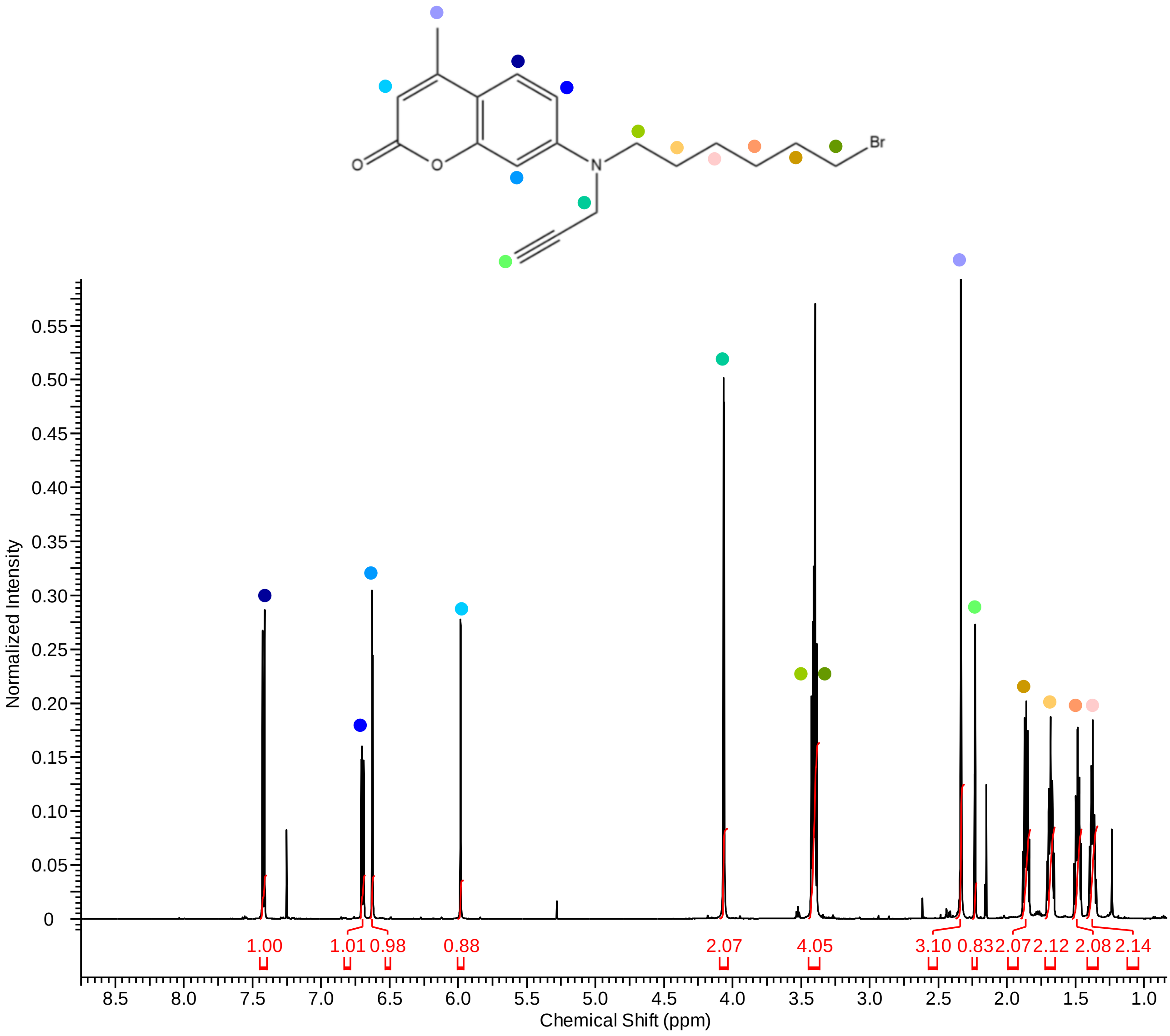

Figure S1. ^{1}H NMR spectrum of 7-((6-bromohexyl)(prop-2-yn-1-yl)amino)-4-methyl-2H-chromen-2-one (**1b**) in $CDCl_3$ (600 MHz, 298 K).

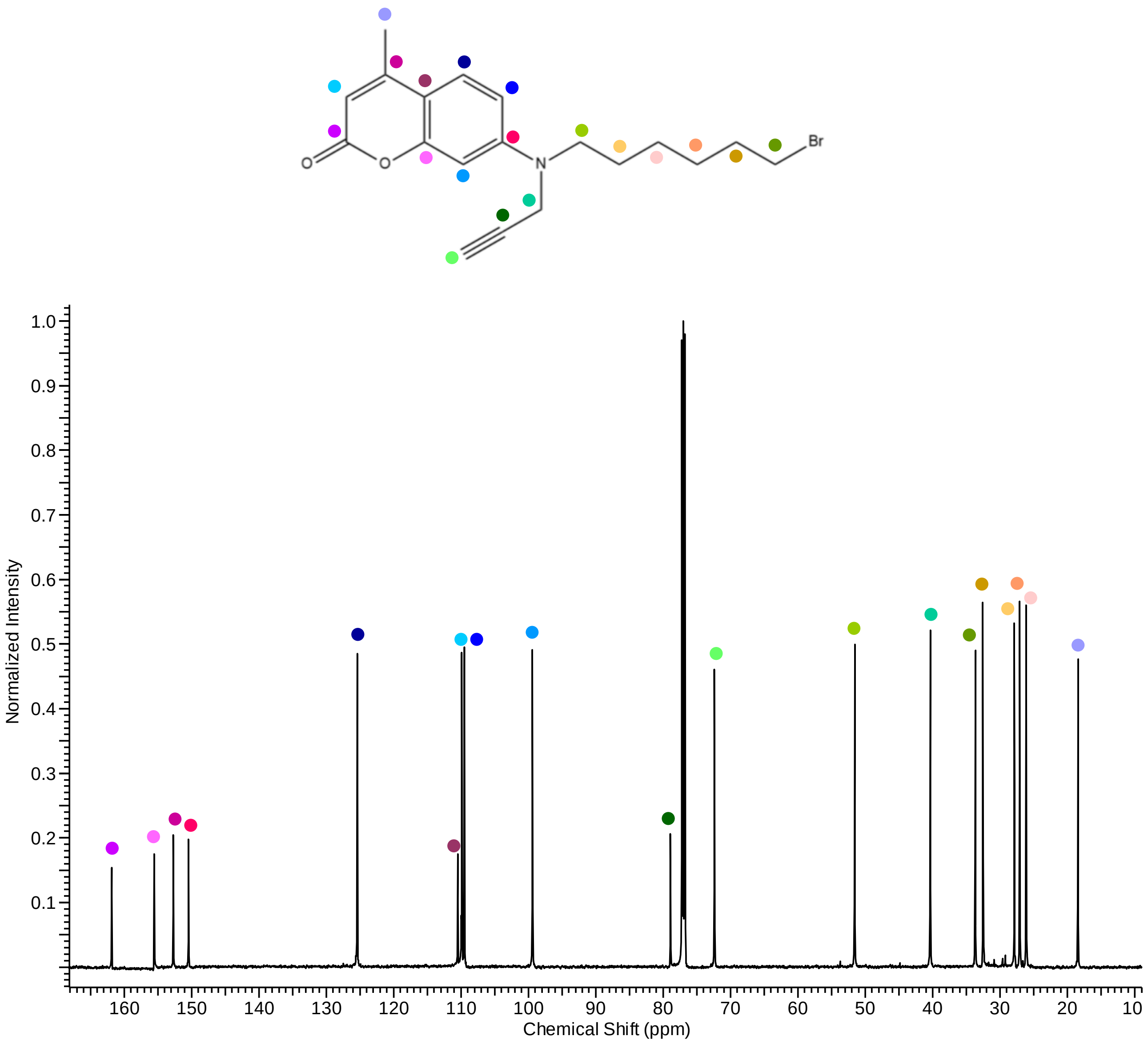

Figure S2. ^{13}C NMR spectrum of 7-((6-bromohexyl)(prop-2-yn-1-yl)amino)-4-methyl-2H-chromen-2-one (**1b**) in $CDCl_3$ (600 MHz, 298 K).

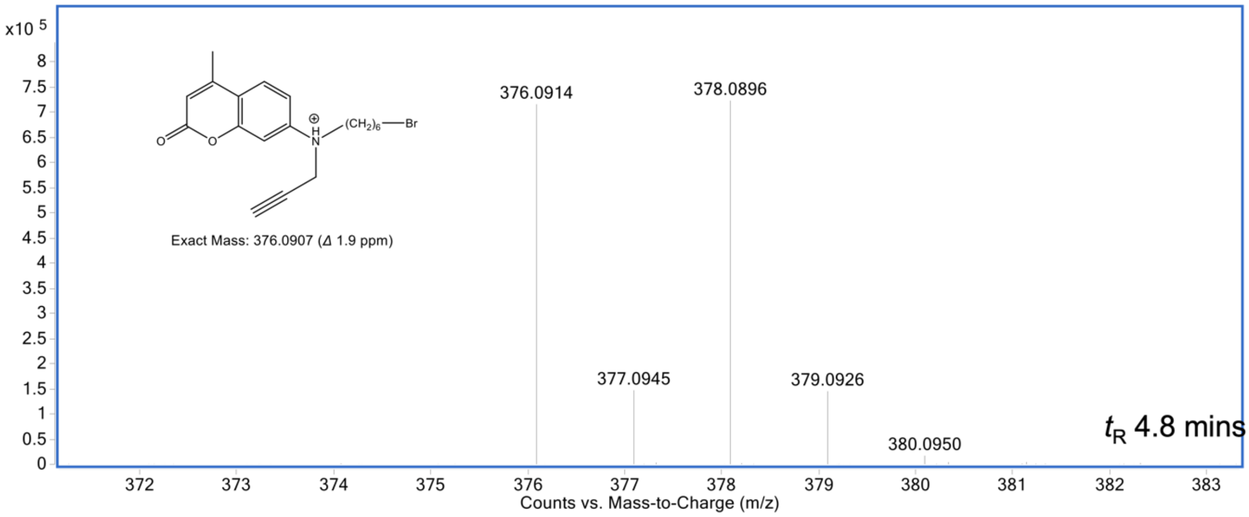

Figure S3. MS spectrum of 7-((6-bromohexyl)(prop-2-yn-1-yl)amino)-4-methyl-2H-chromen-2-one (**1b**).

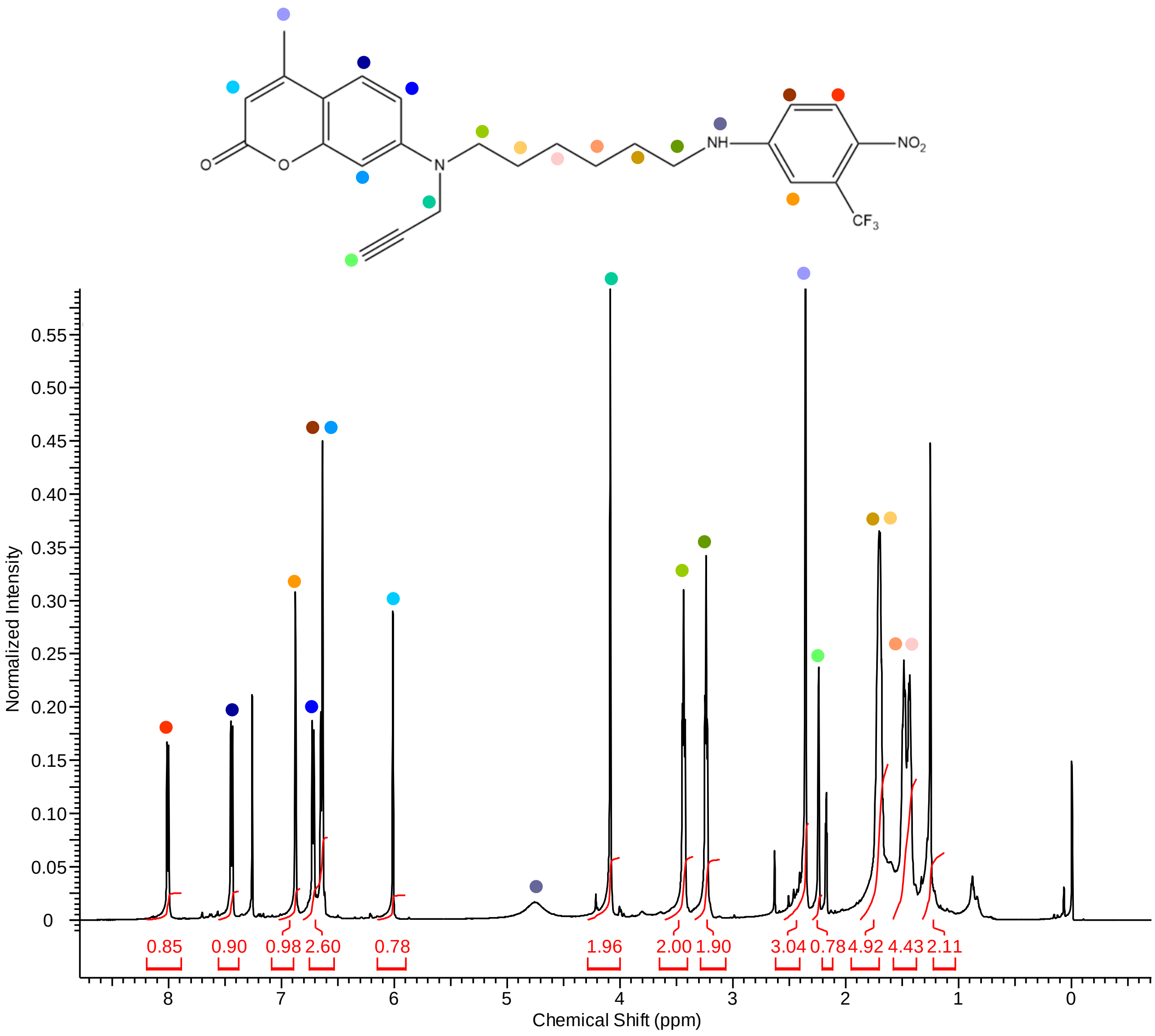

Figure S4. ^{1}H NMR spectrum 4-methyl-7-((6-((4-nitro-3-(trifluoromethyl)phenyl)amino)hexyl)(prop-2-yn-1-yl)amino)-2H-chromen-2-one (**1c**) in $CDCl_3$ (600 MHz, 298 K).

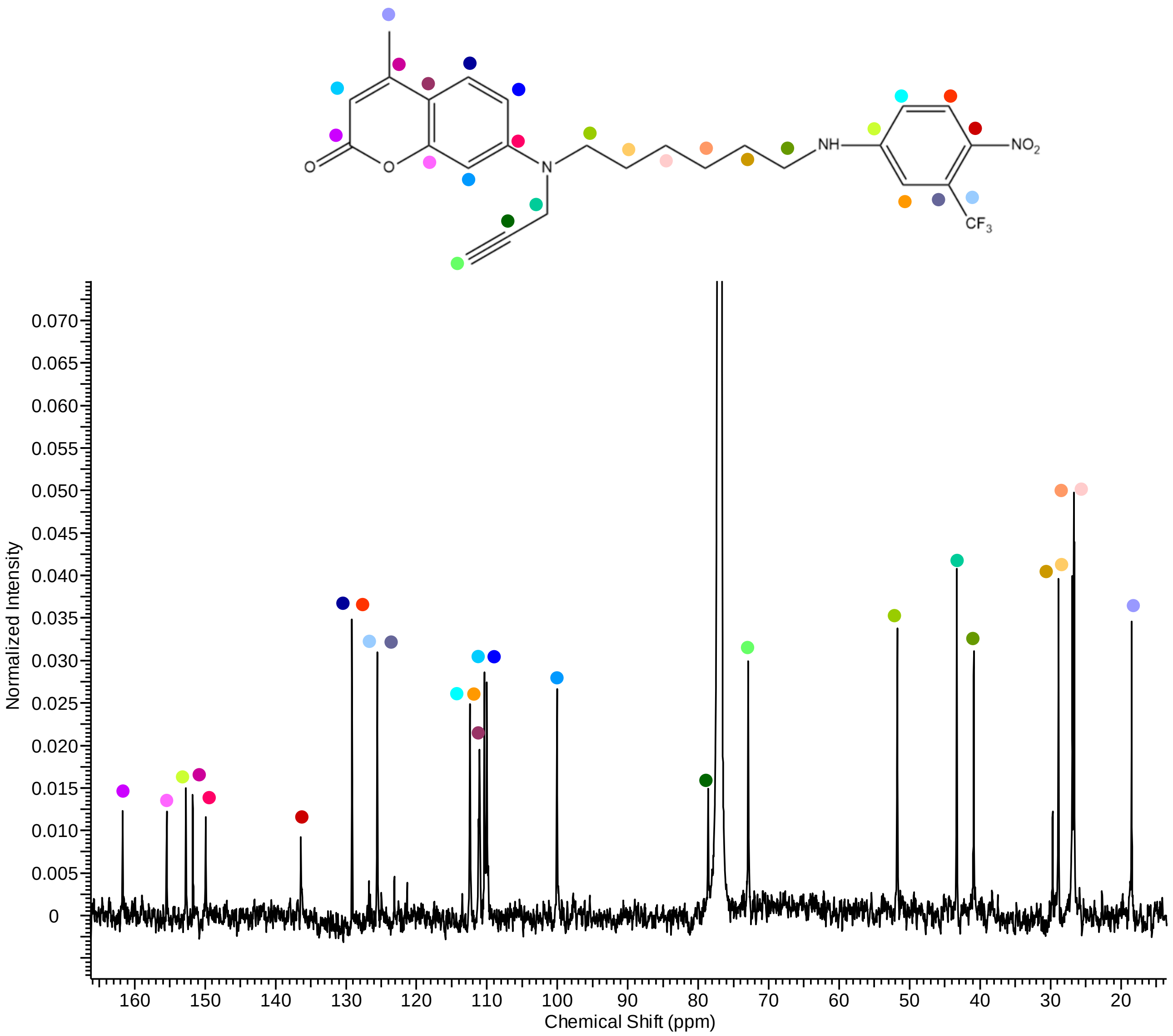

Figure S5. ^{13}C NMR spectrum 4-methyl-7-((6-((4-nitro-3-(trifluoromethyl)phenyl)amino)hexyl)(prop-2-yn-1-yl)amino)-2H-chromen-2-one (**1c**) in $CDCl_3$ (600 MHz, 298 K).

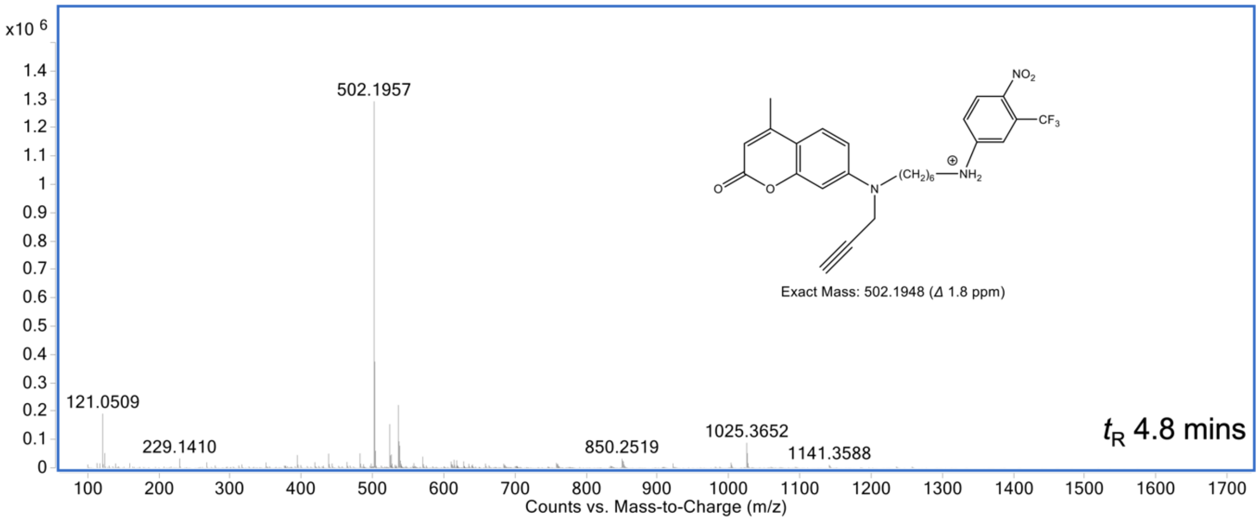

Figure S6. MS spectrum of 7-((6-bromohexyl)(prop-2-yn-1-yl)amino)-4-methyl-2H-chromen-2-one (**1c**).

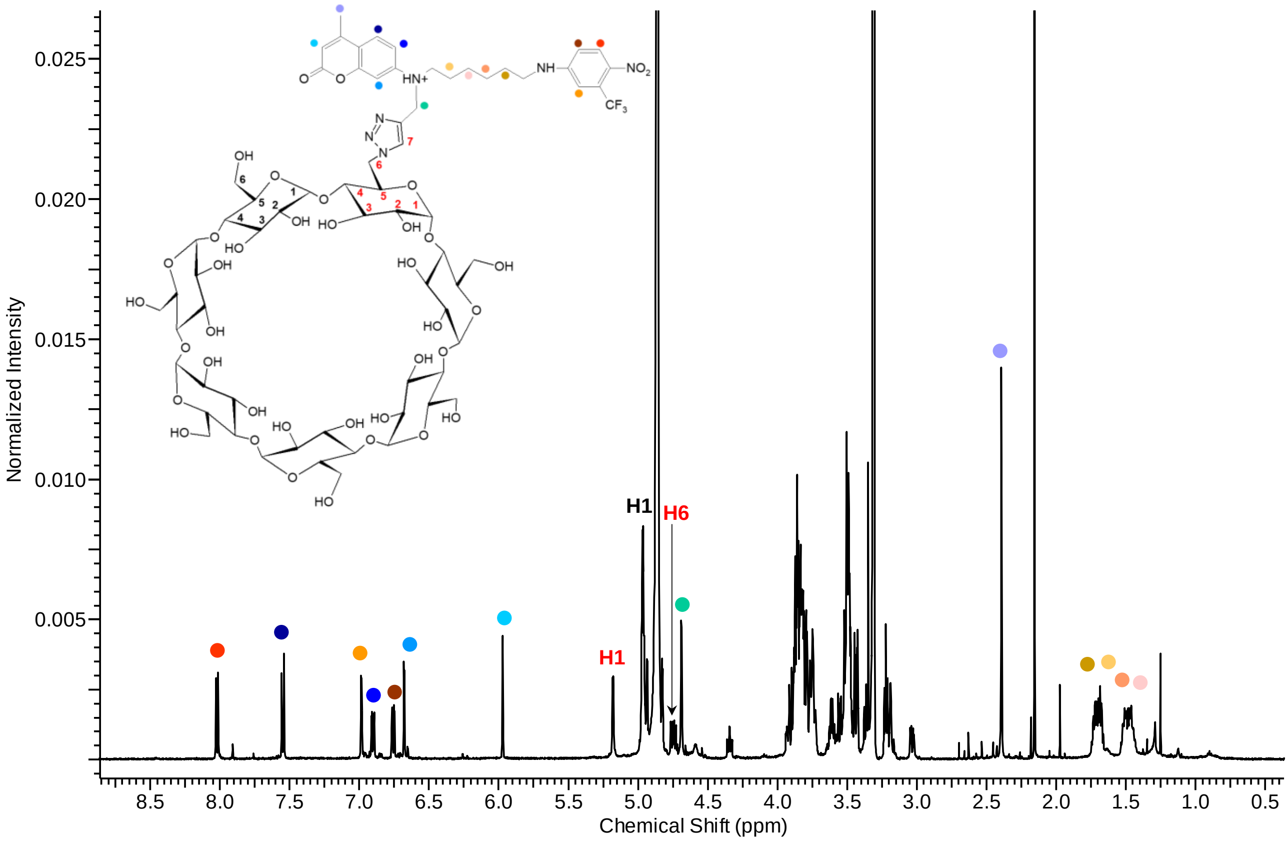

Figure S7. ^{1}H NMR spectrum of **βCD1** in MeOD (600 MHz, 298 K).

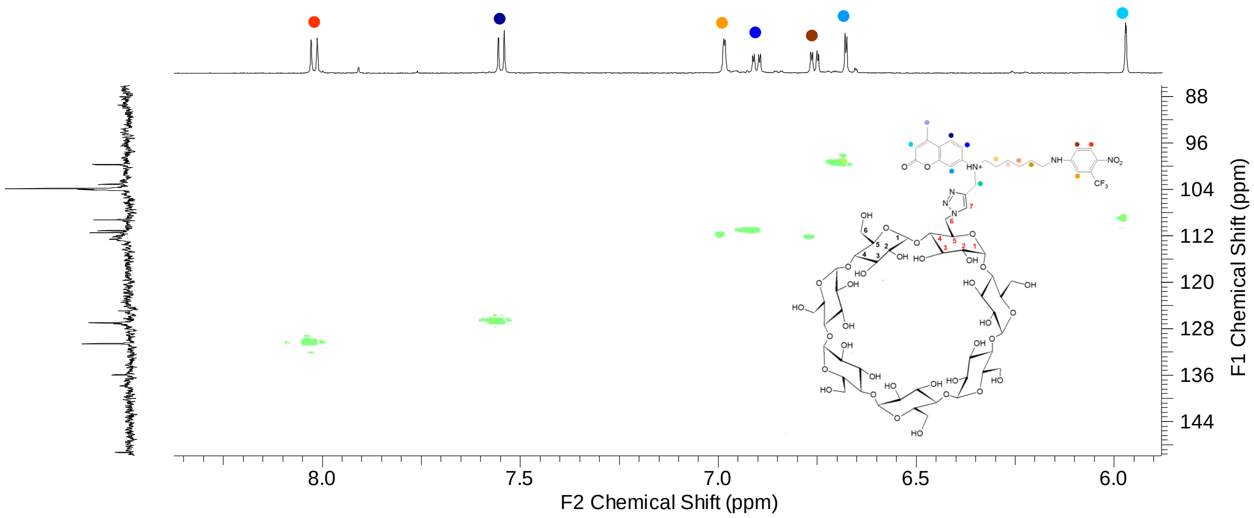

Figure S8. ^{1}H DEPT-edited HSQC spectrum of **βCD1** in MeOD with partial assignment (600 MHz, 298 K) from 8.3 ppm to 5.9 ppm.

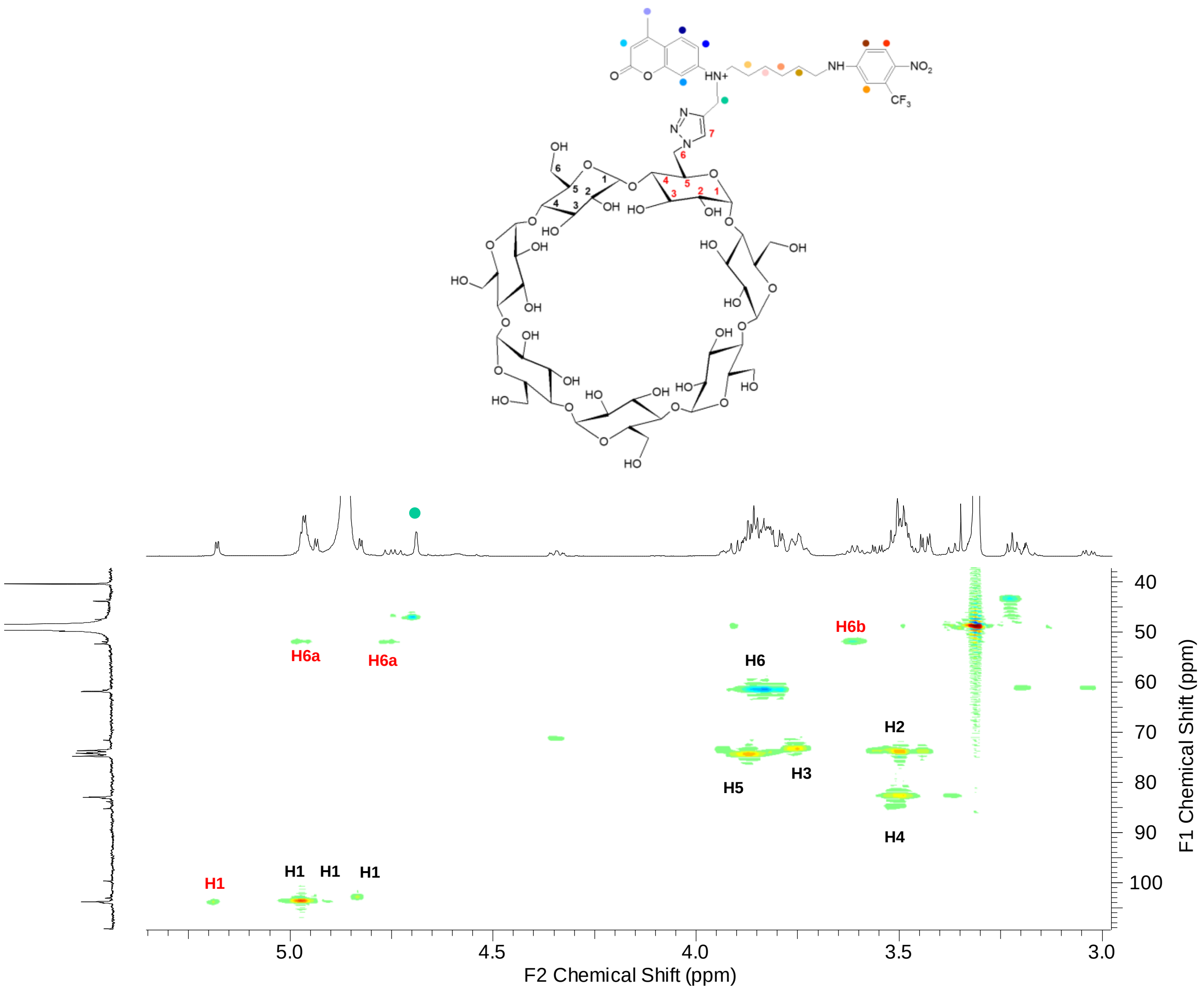

Figure S9. ^{1}H DEPT-edited HSQC spectrum of **βCD1** in MeOD with partial assignment (600 MHz, 298 K) from 5.3 ppm to 3 ppm.

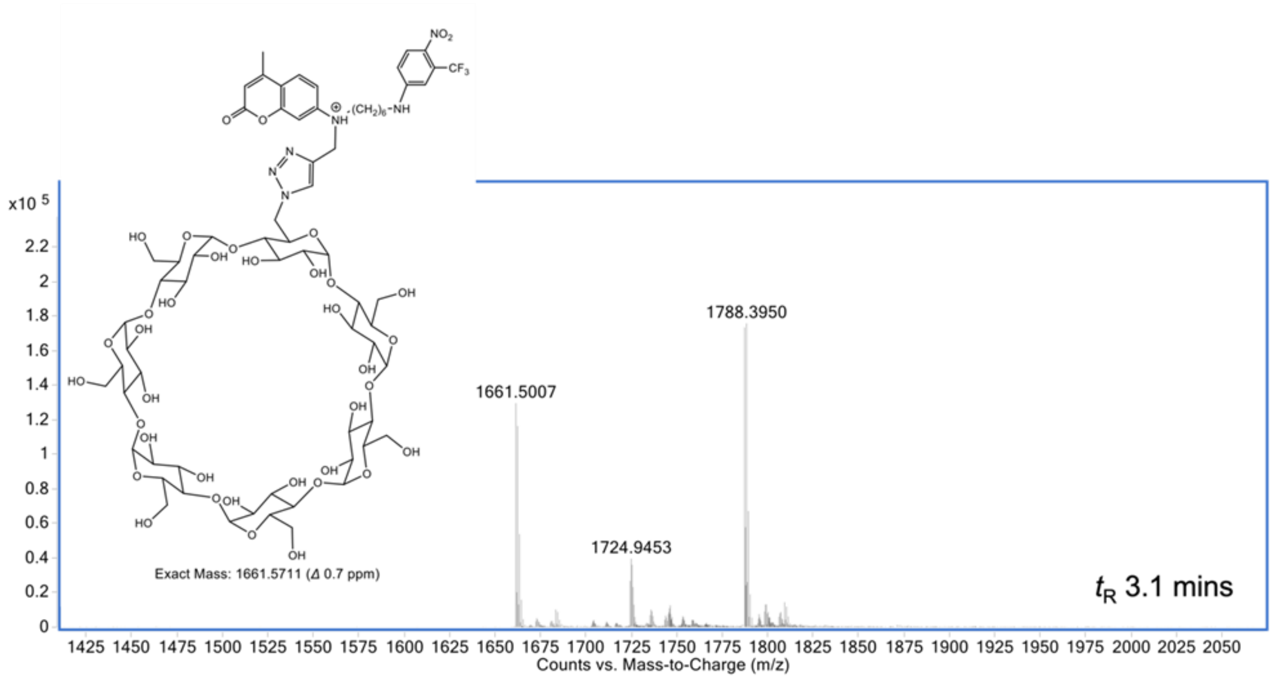

Figure S10. MS spectrum of **βCD1**
